# SGE-Flow: 4D mmWave Radar 3D Object Detection via Spatiotemporal Geometric Enhancement and Inter-Frame Flow

**DOI:** 10.3390/s26051679

**Published:** 2026-03-06

**Authors:** Huajun Meng, Zijie Yu, Cheng Li, Chao Li, Xiaojun Liu

**Affiliations:** 1 Aerospace Information Research Institute, Chinese Academy of Sciences, Beijing 100190, China; menghuajun19@mails.ucas.edu.cn (H.M.); yuzijie21@mails.ucas.edu.cn (Z.Y.); cli@mail.ie.ac.cn (C.L.); lxjdr@mail.ie.ac.cn (X.L.); 2 Key Laboratory of Electromagnetic Radiation and Sensing Technology, Chinese Academy of Sciences, Beijing 100190, China; 3 School of Electronic, Electrical and Communication Engineering, University of Chinese Academy of Sciences, Beijing 100049, China

**Keywords:** 4D millimeter-wave radar, 3D object detection, PointPillars, spatiotemporal enhancement, inter-frame flow

## Abstract

4D millimeter-wave radar provides a promising solution for robust perception in adverse weather. Existing detectors still struggle with sparse and noisy point clouds, and maintaining real-time inference while achieving competitive accuracy remains challenging. We propose SGE-Flow, a streamlined PointPillars-based 4D radar 3D detector that embeds lightweight spatiotemporal geometric enhancements into the voxelization front-end. Velocity Displacement Compensation (VDC) leverages compensated radial velocity to align accumulated points in physical space and improve geometric consistency. Distribution-Aware Density (DAD) enables fast density feature extraction by estimating per-pillar density from simple statistical moments, which also restores vertical distribution cues lost during pillarization. To compensate for the absence of tangential velocity measurements, a Transformer-based Inter-frame Flow (IFF) module infers latent motion from frame-to-frame pillar occupancy changes. Evaluations on the View-of-Delft (VoD) dataset show that SGE-Flow achieves 53.23% 3D mean Average Precision (mAP) while running at 72 frames per second (FPS) on an NVIDIA RTX 3090. The proposed modules are plug-and-play and can also improve strong baselines such as MAFF-Net.

## 1. Introduction

Environmental perception is the foundation of the autonomous driving system, and 3D object detection is a key sub-task of environmental perception [1,2]. While current systems rely heavily on Light Detection and Ranging (LiDAR) [3,4] and cameras, these sensors have a major limitation: their performance degrades significantly in adverse weather like heavy rain, fog, or snow [5]. This limitation has driven increasing interest among researchers in 4D millimeter-wave radar. In addition to its robustness in all weather, 4D radar provides a unique advantage by directly extracting object radial velocity from the Doppler effect. Furthermore, recent hardware improvements have increased its resolution, providing point densities closer to low-beam LiDAR [6].

However, directly applying LiDAR-based algorithms to radar data is difficult. 4D radar point clouds are extremely sparse—often less than 1% of LiDAR’s density—and contain significant noise. Current research generally falls into two categories: accuracy-focused methods like SMURF [7] and MAFF-Net [8], which are slow due to heavy feature extraction; and efficiency-focused designs like RadarPillars [9], which often perform poorly because they ignore geometric distortions caused by ego-motion. A key challenge remains: reconciling a competitive detection accuracy with inference efficiency.

To address these challenges, we propose a lightweight enhancement framework designed specifically for 4D radar. Instead of introducing architectural complexity, our approach—based on a PointPillars [4] baseline—focuses on using physical and geometric insights directly in the feature extraction process. We handle multi-frame “ghosting” artifacts with a Velocity Displacement Compensation (VDC) strategy that aligns points in physical space. To recover vertical information lost during 2D pillarization, we introduce a Distribution-Aware Density (DAD) metric based on statistical dispersion. Most importantly, we address the radar’s inability to measure tangential velocity with a Transformer-based Inter-frame Flow (IFF) module, which identifies motion by analyzing the pillar’s occupancy changes over time.

Our main contributions are summarized as follows:

- To address the tangential velocity indeterminacy, we introduce a Transformer-based Inter-frame Flow (IFF) module. This module identifies latent motion cues by modeling the “Inflow and outflow” of pillar occupancy across frames without requiring explicit velocity measurements.
- We mitigate the vertical resolution deficiency with a Distribution-Aware Density (DAD) metric. By computing statistical moments within each pillar, the metric reconstructs the vertical details often lost during voxelization.
- We resolve “ghosting” artifacts in multi-frame data by a Velocity Displacement Compensation (VDC) strategy. This ensures geometric consistency by aligning features in physical space, rectifying the motion-induced smearing effect for moving targets.
- The proposed framework strikes a superior balance between latency and accuracy, maintaining 72 FPS on an NVIDIA GeForce RTX 3090 GPU. The modular design allows for easy integration into state-of-the-art networks like MAFF-Net [8], demonstrating robust generalization.

## 2. Related Work

### 2.1. 3D Object Detection with 4D Radar Point Clouds

Recent comprehensive surveys [6,10] have systematically summarized the advancements in 4D millimeter-wave radar, emphasizing its robust perception capabilities and all-weather operational characteristics as a solution to the limitations of camera and LiDAR-based systems. Since 4D radar data is structurally similar to LiDAR point cloud data, early algorithms are mainly adapted from established LiDAR frameworks. Point cloud datasets such as VoD [11] and TJ4DRadSet [12] have been important, driving the development of radar-based detection by establishing baselines with LiDAR-based models.

In the LiDAR domain, VoxelNet [3] introduced voxel-based feature extraction but suffered from high computational costs, and PointPillars [4] improved the cost by projecting 3D space into 2D pillars, significantly increasing efficiency. Subsequent works such as PV-RCNN [13] and CenterPoint [14] further optimized detection performance by combining point-voxel features or adopting center-based anchor-free designs. Recognizing this advantage, the 4D radar community has largely adopted voxelization architectures. For instance, RadarPillars [9] introduces self-attention among non-empty pillars to aggregate features belonging to the same object; and RPFA-Net [15] uses self-attention mechanisms to extract global information from radar pillars for better orientation estimation. Departing from these single-view voxelization methods, MVFAN [16] addresses vertical feature deficiency by projecting point clouds into multiple views, including Bird’s Eye View (BEV) and cylindrical coordinates; RadarNeXt [17] proposes a robust detector tailored for 4D radar characteristics, while RCBEVDet [18] explores effective radar-camera fusion strategies in the BEV space.

However, directly applying LiDAR algorithms to 4D radar often produces suboptimal results due to the inherent sparsity and significant noise of radar data [10]. To solve these problems, recent studies have focused on advanced feature enhancement. SMURF [7] addresses density issues by adding a Kernel Density Estimation (KDE) branch to improve sparsity awareness. MAFF-Net [8] proposes a cylindrical denoising assist module to identify keypoints around objects, thereby reducing noise interference. Additionally, SCKD [19] uses cross-modality distillation to transfer knowledge from LiDAR to 4D radar, filtering out noise without reducing inference speed.

### 2.2. Radar Feature Extractor

In pillar-based detection frameworks, the effectiveness of the feature extractor is critical. Addressing the specific characteristics of 4D radar, existing designs primarily focus on distribution feature modeling and velocity information utilization.

The extreme sparsity of single-frame radar data poses a fundamental challenge. Approaches such as RadarMFNet [20] use a modified PointPillars on aggregated multi-frame 4D radar point clouds to alleviate this issue. The 4D radar dataset VoD provides preprocessed single-frame, three-frame, and five-frame data. Furthermore, many methods design specific feature extractors. For instance, DADAN [21] effectively addresses the foreground–background confusion in sparse point clouds by using the unique dynamic information (velocity and intensity) of 4D radar. Similarly, drawing inspiration from the Dual Attention Network [22] in scene segmentation, PillarDAN [23] incorporates a pillar-based dual attention mechanism to capture long-range dependencies in sparse radar data. SMURF [7] designs a density feature by KDE. However, these methods often rely on computationally intensive operations. A key limitation of the PointPillars architecture is the lack of vertical geometric information during the flattening process. To resolve this efficiently, we introduce a lightweight Distribution-Aware Density (DAD) feature that recovers lost structural details with negligible computational cost.

Considering the raw data feature, Doppler velocity gives radar a distinct advantage over LiDAR. RadarPillars [9] demonstrated that decomposing radial velocity boosts performance by aligning features with convolution kernels. Nevertheless, radar’s instantaneous velocity alone is insufficient for complex dynamic scenes. Resolving the limitation that radar cannot measure tangential velocity, we design an Inter-frame Flow (IFF) module inspired by the temporal consistency observed in recent works [5].

While previous methods have improved accuracy, complex additions like those in SMURF and MAFF-Net often compromise latency. We aim to resolve this trade-off by proposing an efficient scheme that simultaneously addresses sparsity and velocity information utilization while ensuring real-time performance.

## 3. Materials and Methods

**Figure 1:**
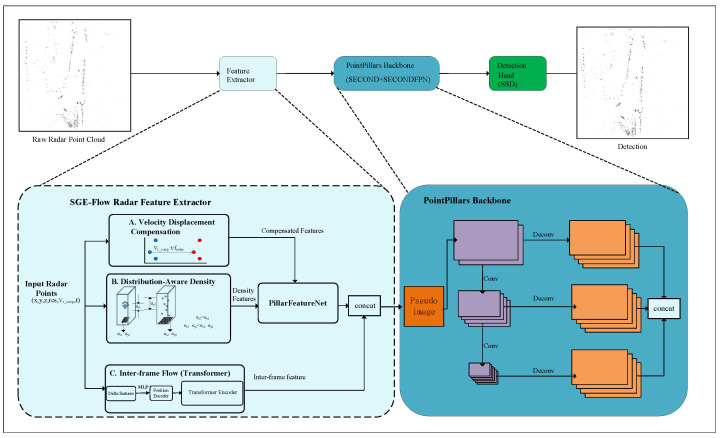
Overview of the proposed 4D radar 3D object detection network architecture. The framework is based on the PointPillars baseline and embeds Velocity Displacement Compensation (VDC), Distribution-Aware Density (DAD), and Inter-frame Flow (IFF) modules. In the VDC module, blue and red circles represent the point clouds before and after compensation, respectively, where $v_{r\_comp}\cdot t/f_{radar}$ denotes the displacement value. The DAD schematic illustrates the density estimation process based on the standard deviation of point distributions. The PointPillars backbone takes the pseudo image as input and follows a standard architecture, including a downsampling branch (purple) and a Feature Pyramid Network (FPN) structure with upsampling via deconvolution (orange) followed by feature concatenation. In the Detection part, red bounding boxes indicate the final 3D object detection results.

We present a streamlined 3D object detection framework, SGE-Flow, architected to maximize the trade-off between algorithmic depth and computational efficiency. As illustrated in Figure 1, our approach confronts the sensor’s physical limitations, specifically sparsity, tangential motion ambiguity, and multi-frame geometric distortions. The framework consists of three parallel Modules: Velocity Displacement Compensation (VDC), Distribution-Aware Density (DAD), and Inter-frame Flow (IFF). Rather than stacking heavy, serial branches, we embed these modules directly into the PointPillars voxelization front-end. This design exploits the natural sparsity of 4D radar, channeling computational power specifically to non-empty pillars.

### 3.1. Challenges of Radar Doppler Features

Unlike LiDAR, which relies on Time of Flight (ToF) for ranging, millimeter-wave radar [11] leverages the Doppler effect to directly measure target velocity. This capability offers an advantage in dynamic scene perception and mitigates the limitations of sparse radar data. While advanced 4D radar hardware may implement internal tracking and filtering to estimate full velocity vectors, the input to point-cloud-based 3D object detectors is typically the direct radial velocity measurements derived from the Doppler effect. However, it also introduces a fundamental physical paradox: radar can effectively measure radial velocity ($v_{r}$), but is blind to tangential motion. Consequently, for targets with significant lateral motion, the radial velocity component fails to describe the true motion state, often leading neural networks to treat the valid data as noise.

**Figure 2:**
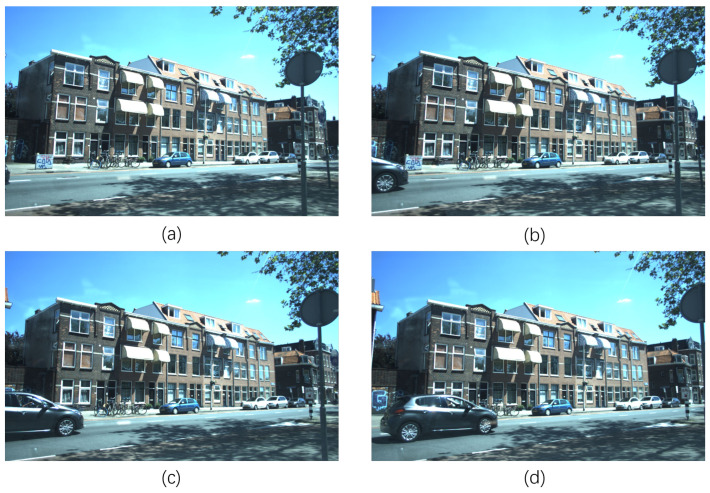
Schematic of consecutive frames for ground-truth velocity estimation: (**a**–**d**) consecutive data frames showing a black vehicle from its initial appearance to full emergence. The interval spans three radar scan cycles, with the total displacement approximately equal to the vehicle’s length.

**Figure 3:**
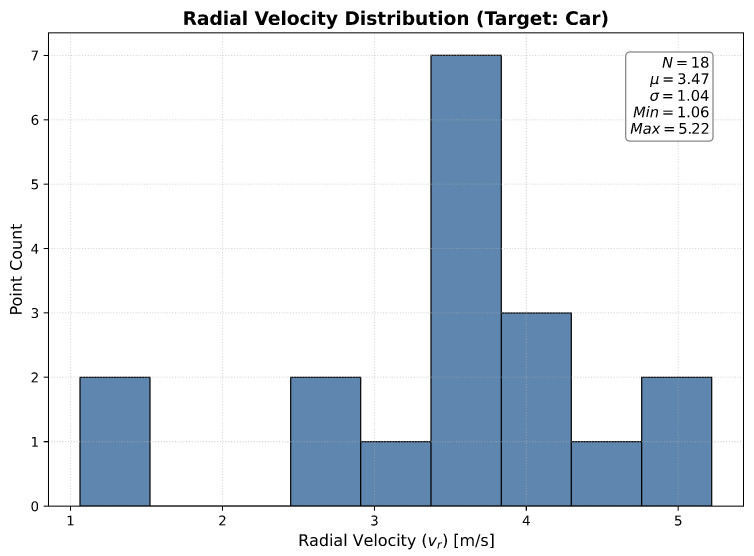
Velocity distribution of radar points localized inside the vehicle bounding boxes shown in Figure 2.

To visualize this, consider the typical lateral motion scenario analyzed in Figure 2, where a vehicle crosses the field of view. Figure 3 shows the velocity distribution of the 4D radar points in the vehicle’s bounding boxes.

The true displacement velocity is defined physically as:

(1) $$v_{\mathrm{gt}}=\frac{\Delta r}{\Delta t}$$

where Δr represents the vehicle’s displacement over time Δt.

Using annotations from the VoD dataset [11] (vehicle length ≈ 4 m, sampling rate 13 Hz), the ground truth velocity is approximately 17.3 m/s. However, statistical analysis of the compensated radial velocity within the vehicle’s bounding box reveals a measured speed of only around 4 m/s—far lower than the actual displacement velocity. This discrepancy highlights how the lack of tangential velocity information causes Doppler data to exhibit noise-like characteristics in lateral motion scenarios.

### 3.2. Velocity Displacement Compensation (VDC)

**Figure 4:**
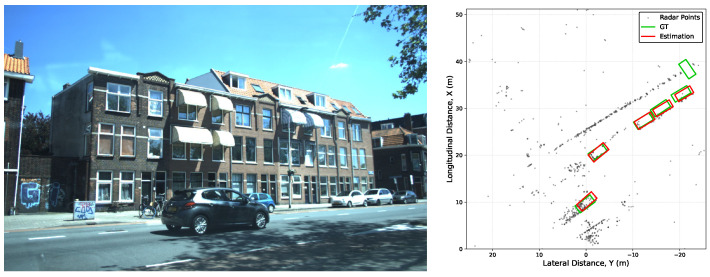
Illustration of the motion-induced smearing effect in a real-world scenario. (**Left**) Camera scene context. (**Right**) Corresponding 4D radar point cloud distribution where the smearing effect extends significantly beyond the ground-truth bounding box boundaries.

While multi-frame accumulation effectively increases the density of sparse radar point clouds, it inevitably introduces motion-induced smearing. Simply superimposing historical frames onto the current coordinate system—compensating only for ego-motion—leaves a trailing “ghosting” artifact along the trajectory of moving targets as shown in Figure 4. Furthermore, the raw velocity data $v_{r}$ lack directional vector information, creating a geometric mismatch with the Cartesian-based convolution operators of Convolutional Neural Networks (CNNs).

Thus, we propose the Velocity Displacement Compensation (VDC) feature and its intuition is straightforward: by recovering the radial location of a point at a past timestamp relative to the current frame, we can align the “ghosts” with the target’s current position. Let the raw radar point cloud be denoted as $P=\{p_{i}|i=1,\ldots ,N\}$, where each point $p_{i}$ carries a feature vector $f_{i}={\left[x_{i},y_{i},z_{i},v_{r,i},v_{r,\mathrm{comp}},t_{i}\right]}^{T}$, with $v_{r,i}$ representing the Doppler radial velocity, $v_{r,\mathrm{comp}}$ representing the radial velocity after compensating for the ego-motion of the sensor platform, and $t_{i}\in \left\{0,\ldots ,T-1\right\}$ the timestamp index.

To correct geometric distortion, we first calculate the unit direction vector $u_{i}$ and time lag $\Delta \tau_{i}$:

(2) $$u_{i}=\frac{{\left[x_{i},y_{i},z_{i}\right]}^{T}}{\sqrt{x_{i}^{2}+y_{i}^{2}+z_{i}^{2}}},\;\Delta \tau_{i}=\frac{t_{i}}{f_{\mathrm{radar}}}$$

where $x_{i},y_{i},z_{i},t_{i}$ is the raw data of the point cloud, and $f_{\mathrm{radar}}$ is the radar sampling frequency.

From this, we derive the spatial displacement vector $\Delta d_{i}$ driven by the target’s motion:

(3) $$\Delta d_{i}={\left[\Delta x_{i},\Delta y_{i},\Delta z_{i}\right]}^{T}=v_{r,\mathrm{comp}}\cdot \Delta \tau_{i}\cdot u_{i}$$

This spatial displacement is calculated by projecting the compensated radial velocity into 3D Cartesian coordinates along the direction vector $u_{i}$ over the duration $\Delta \tau_{i}$.

The final enhanced feature is defined as ${\hat{f}}_{i}=\left[f_{i},\Delta d_{i}\right]$. This explicit displacement modeling effectively projects the radial scalar $v_{r}$ into Cartesian space, reconciling the misalignment between polar velocity and Euclidean convolution kernels.

### 3.3. Distribution-Aware Density (DAD)

Density features carry a significant impact on 3D object detection and extensive research has been conducted to exploit this information [24]. Standard PointPillars voxelization [4] relies on lossy compressional max-pooling for feature aggregation. This operation strips away many geometric details, particularly along the vertical (Z-axis). Furthermore, conventional voxelization partitions point clouds into fixed-size grids. This rigid quantization implies that any points within a single pillar—regardless of whether they are dispersed or tightly clustered—will yield an identical density feature as long as their cardinality remains constant, exacerbating foreground–background confusion [25].

We propose the Distribution-Aware Density (DAD) feature. Our core intuition is to treat the various statistical moments of the point cloud within each pillar as a representative descriptor of its spatial geometric distribution.

For any non-empty Pillar $P_{j}$, let $I_{j}=\left\{k|p_{k}\in P_{j}\right\}$ be the set of point indices, and $N_{j}=\left|I_{j}\right|$ the point count. We describe the local micro-structure by calculating the geometric center $\mu_{j,c}$ and standard deviation $\sigma_{j,c}$ in spatial dimensions c∈{x,y,z}:

(4) $$\mu_{j,c}=\frac{1}{N_{j}}\sum_{k\in I_{j}}p_{k,c}$$

(5) $$\sigma_{j,c}=\sqrt{\frac{1}{N_{j}-1}\sum_{k\in I_{j}}{\left(p_{k,c}-\mu_{j,c}\right)}^{2}}$$

Recognizing that the BEV plane (XY) and height direction (Z) carry distinct semantic weights, we decouple the density features into planar distribution $D_{xy}$ and height dispersion $D_{z}$, weighted by the point count $N_{j}$:

(6) $$D_{xy}=\mathrm{Norm}\left(\frac{N_{j}}{\sigma_{x}\cdot \sigma_{y}}\right)$$

(7) $$D_{z}=\mathrm{Norm}\left(\frac{N_{j}}{\sigma_{z}}\right)$$

where Norm(·) denotes the standard Z-score normalization (i.e., (x−μ)/σ) applied to features within each pillar to stabilize numerical ranges. $D_{xy}$ captures the horizontal occupancy density, while $D_{z}$ compensates for the vertical information compressed by pillarization, enabling the network to distinguish between volumetric targets (like pedestrians) and planar clutter.

### 3.4. Inter-Frame Flow (IFF)

Although VDC exploits velocity information, a critical gap remains: the radar’s inability to measure tangential velocity. To recover these latent motion cues, we introduce the Inter-frame Flow (IFF) module, a Transformer-based architecture [26]. The utilization of sequence learning to capture complex spatiotemporal dependencies has also demonstrated effectiveness in other dynamic perception tasks, such as pose forecasting [27].

**Figure 5:**
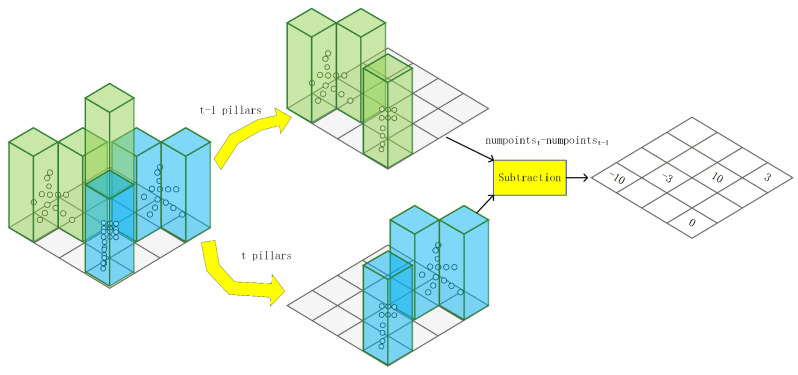
Illustration of the Inter-frame Flow (IFF) calculation method. The green and blue pillars represent the point cloud distributions at time t−1 and *t*, respectively. By computing the difference between adjacent frames, the resulting positive, zero, and negative values denote three states: inflow (target entering), static (no movement), and outflow (target exiting) within the pillar.

Our core insight is that for a moving target, the point cloud traverses different pillars across consecutive time frames, resulting in positive, negative, or zero values when computing the difference between adjacent frames. For instance, an object moving from Pillar A to Pillar B induces a decrease in point count in Pillar A (outflow) and a corresponding increase in Pillar B (inflow). This inter-pillar state difference implicitly encodes the target’s motion flow. In contrast, pillars occupied by stationary objects remain consistent across frames, leading to a zero-value difference. This process is visualized in Figure 5, where the pillars of the previous and current frames are color-coded in green and blue, respectively. Based on the inter-frame difference, pillars representing the outflow of moving targets yield negative values, those representing inflow result in positive values, and stationary pillars result in zero. By capturing these temporal transitions, the network can effectively infer the underlying kinematic characteristics from the discretized spatial grid.

**Figure 6:**
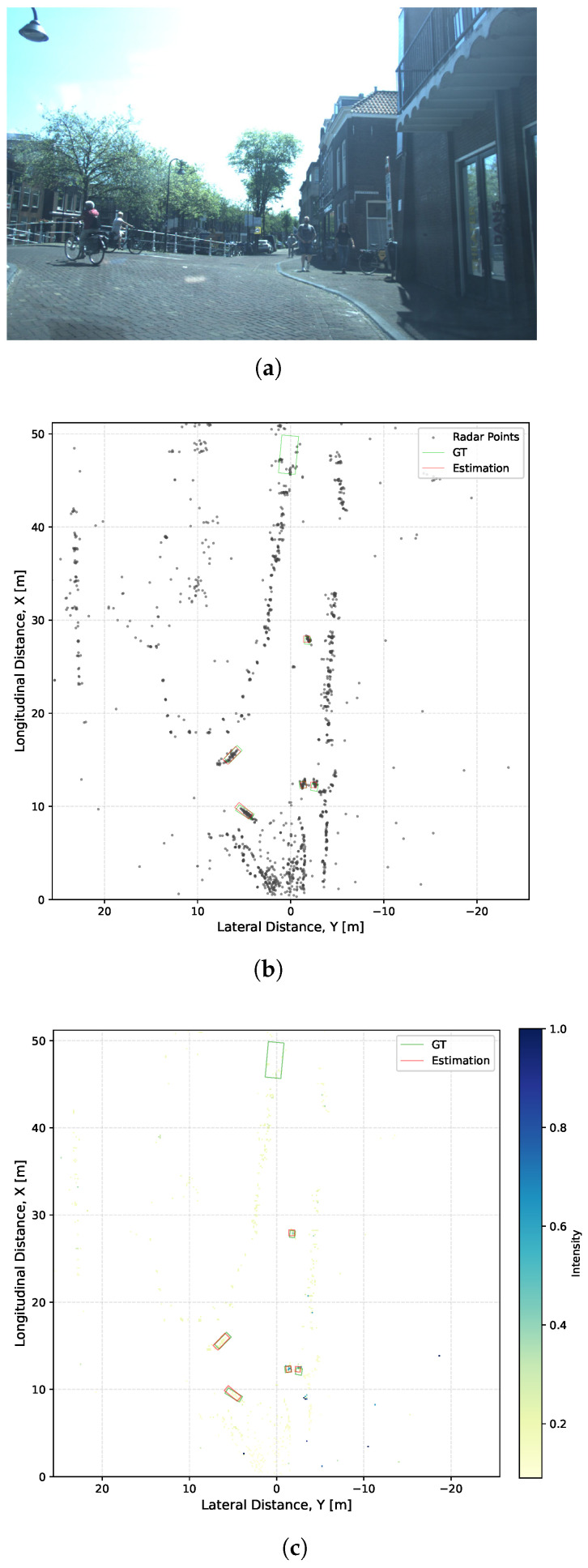
Visualization of the feature enhancement effect of the IFF module. The red and green boxes represent the object Estimation and Ground Truth, respectively. (**a**) Camera image providing the scene context; (**b**) BEV distribution of 4D radar point cloud; and (**c**) IFF-based feature enhancement map with object estimations.

A qualitative visualization of the IFF module’s impact is presented in Figure 6. While Figure 6a displays the raw scene, Figure 6b provides the BEV visualization of estimated and ground-truth bounding boxes. Figure 6c illustrates the L2-norm of the feature residuals induced by the IFF module, where deeper blue intensities signify a more pronounced feature enhancement. As highlighted by the red circles, the regions corresponding to cyclists and pedestrians exhibit significantly higher activation compared to the ambient environment. Although some isolated dark blue pixels appear elsewhere—likely stemming from inherent sensor noise—these discrete activations do not correspond to coherent target structures and thus exert no adverse influence on the final detection performance. This observation confirms the module’s robustness in selectively isolating kinematic signatures from potential clutter.

#### 3.4.1. Temporal Difference Feature Construction

We treat each Pillar $P_{j}$ as a temporal vessel. For timestamps t∈{0, 1,…, 4}, we first aggregate the point-wise features. Let $N_{j,t}$ denote the point count at time *t*. We derive the mean compensated radial velocity ${\bar{v}}_{t}$ and mean radar reflectivity ${\bar{r}}_{t}$:

(8) $${\bar{v}}_{t}=\frac{1}{N_{j,t}}\sum_{p\in P_{j,t}}v_{r\_comp}^{\left(p\right)}$$

(9) $${\bar{r}}_{t}=\frac{1}{N_{j,t}}\sum_{p\in P_{j,t}}{\mathrm{RCS}}^{\left(p\right)}$$

where $v_{r\_comp}^{\left(p\right)}$ and ${\mathrm{RCS}}^{\left(p\right)}$ are the compensated radial velocity and Radar Cross Section (RCS) of point *p*.

To capture the local flow, we construct the first-order Feature Gradient between adjacent steps:

(10) $$\Delta N_{t}=N_{t+1}-N_{t}$$

(11) $$\Delta {\bar{v}}_{t}={\bar{v}}_{t+1}-{\bar{v}}_{t},\;\Delta {\bar{r}}_{t}={\bar{r}}_{t+1}-{\bar{r}}_{t}$$

where $\Delta N_{t}>0$ signals an object entering the pillar, while $\Delta N_{t}<0$ indicates departure.

These difference quantities $\left[\Delta N_{t},\Delta {\bar{v}}_{t},\Delta {\bar{r}}_{t}\right]$ form the raw sequence describing the local flow dynamics.

#### 3.4.2. Transformer Temporal Aggregation

To model global temporal correlations, we map these difference features to a high-dimensional embedding vector $h_{flow}$ by a Multi-Layer Perceptron (MLP), injecting Positional Encoding (PE) to preserve spatial context:

(12) $${\mathrm{PE}}_{\left(pos,2i\right)}=\sin\left(pos/10000^{2i/d_{model}}\right)$$

(13) $${\mathrm{PE}}_{\left(pos,2i+1\right)}=\cos\left(pos/10000^{2i/d_{model}}\right)$$

where the pos denotes the specific index of each pillar in the flattened sequence, while $i\in [0,d_{model}/2)$ represents the dimension index. These equations represent standard sine and cosine positional encodings, consistent with the Transformer architecture [26].

Introducing such sinusoidal modulation is essential to break the permutation invariance inherent in the Transformer encoder, thereby allowing the network to perceive the spatial relative positions of pillars in the pseudo-image.

The input feature $H_{in}$ is then constructed by adding the position encodings to the high-dimensional embedding vector $h_{flow}$:

(14) $$H_{in}=h_{flow}+{\mathrm{PE}}_{x}+{\mathrm{PE}}_{y}$$

This sequence is then fed into the Transformer Encoder to distill global temporal context, followed by a global residual connection and layer normalization:

(15) $$H_{out}=\mathrm{LayerNorm}\left(h_{flow}+\mathrm{TransformerEncoder}\left(H_{in}\right)\right)$$

Finally, the temporal flow feature $H_{out}$ and the static geometric feature $F_{pillar}$ from the Pillar Encoder are concatenated. This results in a pseudo-image feature map rich in both spatiotemporal and geometric information, serving as the input for the backbone network.

### 3.5. Other Parts of the Network

We adopt the SECOND network [28] as the 2D backbone, utilizing a simplified PointNet [29] as the initial pillar encoder to efficiently extract scene-level features. As shown in the standard configuration, the backbone contains three convolutional blocks with layer depths of {3, 5, 5}, downsampling strides of {2, 2, 2}, and output channel counts of {64, 128, 256}. To fuse multi-scale features, we adopt SECOND-FPN (Feature Pyramid Network) as the Neck. It upsamples features from different levels (strides {1, 2, 4}) and concatenates them into a unified representation. Finally, we use the standard Anchor3DHead for detection. The detection head estimates the class score, 3D box regression residuals, and direction estimation of targets based on predefined Anchors.

Loss Function: The training of the network uses a multi-task loss function $L_{total}$ for end-to-end optimization. Specifically, we use Focal Loss [30] with parameters γ=2.0 and α=0.25 to calculate the classification loss ($L_{cls}$), Smooth L1 Loss to calculate the bounding box regression loss ($L_{bbox}$), and Cross-Entropy Loss to calculate the direction classification loss ($L_{dir}$). The total loss $L_{total}$ is defined as a weighted linear combination of these components:

(16) $$L_{total}=\beta_{cls}L_{cls}+\beta_{box}L_{box}+\beta_{dir}L_{dir}$$

where $\beta_{cls}$, $\beta_{box}$, and $\beta_{dir}$ are balancing coefficients, set to 1.0, 2.0, and 0.2, respectively, to harmonize the different loss magnitudes.

## 4. Results and Discussion

### 4.1. Datasets and Evaluation Metrics

To verify the effectiveness of the proposed method, we conducted extensive evaluations on the View-of-Delft (VoD) dataset [11]. The dataset contains 8693 synchronized frames collected in complex urban driving scenarios. It provides 64-line LiDAR, stereo camera, and 3+1D millimeter-wave radar data. In this work, we present a pure 4D-radar-based detection framework; the LiDAR data are utilized solely for generating ground-truth labels and providing accuracy benchmarks during evaluation, while the inference process relies exclusively on 4D radar point clouds. Given the sparsity of radar point clouds, we follow the standard settings of existing research. We use five-frame accumulated (five scans) radar point clouds as network input and conduct performance tests on the officially divided validation set. Experiments use Average Precision (AP) and Mean Average Precision (mAP) as the main evaluation metric, evaluating the detection performance of three categories: Car, Pedestrian, and Cyclist in the Entire Annotated Area and Driving Corridor, respectively. In addition, random flip, global scaling, and global rotation were used as data augmentation strategies during training. The robustness of the model was further improved through input data normalization.

The model proposed in this paper is built based on the MMDetection3D framework. We chose PyTorch 1.13.0 as the primary development library due to its dynamic computational graph and flexible debugging capabilities, which are essential for prototyping complex spatiotemporal feature extraction modules. For hardware acceleration, the CUDA 11.7 environment is utilized to leverage the massively parallel processing power of NVIDIA GPUs. This setup is critical for efficiently handling computationally intensive operations, such as large-scale point cloud voxelization and Transformer-based temporal attention, thereby ensuring the real-time inference performance (72 FPS) required for autonomous driving. All experiments were completed on the Ubuntu 20.04 operating system (Canonical Ltd., London, UK) with eight NVIDIA RTX3090 (24 GB) GPUs (NVIDIA Corporation, Santa Clara, CA, USA). The inference speed in Table 1 was measured by a single NVIDIA RTX3090 (24 GB) with a batch size of 1.

In terms of network parameter settings, we set the detection range of the point cloud to X∈[0,51.2] m, Y∈[−25.6,25.6] m, Z∈[−3,2] m. To balance detection accuracy and inference speed, the voxel size is set to [0.16,0.16,4] m, and the final generated pseudo-image resolution is 320×320. The training process uses the AdamW optimizer with a weight decay of 0.05. The learning rate adjustment adopts a strategy combining Warmup and Cosine Annealing. The model is trained for a total of 120 epochs, with a warm-up period spanning the first 5 epochs, the peak learning rate set to 0.006, and the Batch Size set to 16. In addition, we conducted dedicated ablation studies on the use of the radial velocity ($v_{r}$) and timestamps (*t*) to determine the optimal feature input combination.

### 4.2. Comparison of with State-of-the-Art (SOTA)

Table 1 details the quantitative landscape, positioning our method against existing state-of-the-art (SOTA) radar detection networks. Our model establishes a strong baseline, outperforming the majority of competing methods in overall 3D detection. MAFF-Net relies on a computationally heavy clustering generation mechanism to refine fine-grained features, whereas our approach prioritizes architectural efficiency.

Despite this, our model remains highly competitive in detecting rigid targets like cars and cyclists. Crucially, within the Driving Corridor area (RoI)—the region most vital for immediate autonomous driving safety—our model exhibits robust performance, underscoring its reliability in perceiving core close-range threats.

Efficiency is not merely a metric but a core design principle of our framework. As shown in Table 1, our SGE-Flow achieves a real-time inference speed of 72 FPS on an RTX 3090, which is significantly faster than high-performance yet computationally expensive models like MAFF-Net [8] (28.7 FPS). While MAFF-Net reaches higher accuracy, its heavy feature extraction pipelines may hinder deployment in high-speed autonomous driving scenarios where latency is critical. By embedding DAD and IFF features directly into the voxelization front-end, SGE-Flow ensures that computational resources are focused on non-empty pillars, maintaining a superior balance between latency and precision.

To further demonstrate the versatility of the proposed modules, we conducted a plug-and-play (PnP) validation study by integrating the IFF module into the state-of-the-art MAFF-Net architecture (denoted as “MAFF-Net + Ours (IFF)”). We specifically selected IFF for this cross-model validation because it addresses the most fundamental physical constraint—the lack of tangential velocity—that persists across different radar detection backbones. The results indicate that adding IFF boosts the overall mAP of MAFF-Net to 55.00%, achieving a significant +7.70% AP gain for cars in the Driving Corridor. This consistent improvement proves that IFF can serve as a universal enhancement for radar temporal modeling, irrespective of the underlying feature extraction complexity.

### 4.3. Ablation Studies

We conducted a detailed series of ablation experiments on the VoD validation set in Table 2. Our results demonstrate a synergistic effect: when Velocity Displacement Compensation (VDC), Distribution-Aware Density (DAD), and Inter-frame Flow (IFF) are introduced at once, the model achieves its greatest performance, increasing Entire-Area mAP by 5.38% over the baseline.

We initiated our work by actively utilizing the foundational architecture of PointPillars to establish the initial baseline configuration for comparison. The empirical results from Experiment 2 clearly show that the deliberate integration of the VDC module provided a measurable boost of 2.99% in mAP across the entire evaluation area. Within this recorded overall gain, it was the categories of Pedestrians and Cyclists that manifested the most substantial degree of performance enhancement, achieving resultant increases of 4.42% and 4.20%, respectively. When concentrating the analysis specifically on the defined driving area, the performance improvement for pedestrians was registered as the most significant, reaching approximately 8.38%. The performance can be attributed to the fact that both pedestrians and cyclists are consistently engaged in continuous motion, a state that allows them to derive substantially greater benefit from the mechanisms responsible for displacement compensation, but the Vehicle category includes both moving cars and those are parked along the roadside.

Experiment 3 indicates that subsequent to the formal incorporation of the DAD module, only the Cyclist category demonstrated a statistically meaningful performance increase, which was approximately 1.76% across the entire spatial area. We attribute this performance gain not only to the derived point cloud density descriptors but also to the vertical distribution cues recovered by the DAD module. In contrast to vehicles and pedestrians, cyclists exhibit a unique vertical profile—characterized by a higher central elevation and a narrower horizontal span—a geometric detail that DAD effectively reconstructs by modeling height dispersion despite 2D pillarization.

Experiment 4 indicates that the IFF module ultimately yielded a perceptible rise of 3.59% for pedestrians and a subsequent increase of 2.76% for cyclists in the full area, thereby making a formal contribution to an overall mAP growth measured at 2.09%. In the driving area, the performance enhancements for pedestrians and cyclists were at 2.97% and 2.52%, respectively, accompanied by an mAP gain of 2.11%. According to this measure, this module exerts the most pronounced impact among all proposed modules. As targets frequently in a state of motion within road scenes, pedestrians and cyclists derive greater gains from IFF due to IFF module’s inherent capability to effectively extract underlying motion-related features.

Secondly, we explored the impact of data augmentation strategies on the physical consistency of radar velocity features (as shown in Table 3). Radar radial velocity $v_{r}$ is a physical quantity highly coupled with the observation angle; previous studies have cautioned that the migration of LiDAR-based data augmentation techniques to 4D radar point clouds must be approached with prudence [11]. Experimental results show that when using $v_{r}$ and $v_{r,\mathrm{comp}}$, although Object Translation and Object Rotation increase the spatial diversity of data, they only bring a slight mAP improvement of 0.24% and 0.13%. This finding highlights a critical nuance in radar perception: while translation generally improves generalization in LiDAR or image domains, in radar, it risks destroying the physical constraints of radial velocity. Specifically, simply translating an object without re-computing its radial velocity components leads to a physical mismatch between the target’s new position and its Doppler signature, introducing noise that offsets the benefits of data diversity. In contrast, Global Rotation preserves the relative geometric relationships and physical consistency of velocity components, significantly increasing mAP to 51.04% (an increase of 1.80%). Furthermore, we observe that when we choose to rely solely upon the compensated radial velocity ($v_{r,\mathrm{comp}}$) as input, consciously setting aside the raw $v_{r}$—a feature which is highly susceptible to contamination from the ego-motion of the sensor platform—the overall model performance peaks notably at 53.23%. This suggests that $v_{r,\mathrm{comp}}$ possesses superior rotational invariance and serves as a more robust motion feature. Therefore, our final strategy relies exclusively on $v_{r,\mathrm{comp}}$ as velocity input, utilizing Global Rotation as the primary geometric augmentation technique.

Since the VoD dataset [11] uses five-frame accumulated point clouds to alleviate sparsity, introducing effective temporal identifiers is crucial for distinguishing historical frame information and understanding target motion states. We compared two temporal information encoding methods in Table 4: directly concatenating raw timestamps (*t*) vs. only using the Inter-frame Flow (IFF) proposed in this paper. Experimental results show that IFF provides superior feature representational capacity compared to discrete time markers. Specifically, under the configuration based on velocity compensation features (VDC), after replacing the input from raw timestamp *t* to the IFF module, the Entire-Area mAP and Driving Corridor mAP increased by +0.62% and +1.05%; under the configuration based on DAD, the IFF module also brought performance gains, increasing Entire-Area mAP by +0.83%. This consistent improvement strongly proves that the IFF module extracted richer motion semantics than simple timestamps by explicitly modeling the temporal correlation between pillars.

We further investigated the potential of combining both features. However, experimental results indicate that re-introducing the raw timestamp *t* alongside the IFF module does not yield cumulative performance gains; instead, it leads to a slight degradation. Consequently, our final configuration retains only the IFF feature and discards the raw timestamp *t*.

In terms of inference efficiency, although the introduction of the Transformer structure increases the computational overhead, resulting in a decrease in inference speed (for example, dropping from 96.1 FPS to 74.7 FPS in the VDC group), the model still maintains a high real-time performance of over 70 FPS. This indicates that the IFF module exchanges acceptable computational cost for better detection accuracy and is a more efficient and robust temporal representation method than the raw timestamp.

The IFF module uses a transformer to model the correlation between pillars. The hyperparameter layer num also has a significant impact on performance. The results obtained using different numbers are shown in Table 5. Based on the results, we comprehensively consider selecting layer num as 2.

The architectural depth of the Transformer encoder that is situated within the IFF module demonstrably exerts a substantial and critical impact upon the overall system performance. We systematically undertook an evaluation of the hyperparameter *L*, which corresponds to the total number of layers, testing it across the specific range of L∈{1, 2, 3}. The results derived from these controlled experiments unequivocally indicate that the setting of L=2 is the specific configuration that successfully achieves the maximally optimal performance level. Although the action of incrementally increasing the layer count from a value of 1 to 2 effectively enhances the representational capacity of the extracted features, the subsequent act of further extending this count to L=3 was observed to bring about a slight, measurable degradation in overall performance. This phenomenon is largely and logically attributable to the onset of over-parameterization, a condition that becomes readily apparent when operating with the inherently sparse data structure characteristic of 4D radar point clouds.

### 4.4. Qualitative Analysis

**Figure 7:**
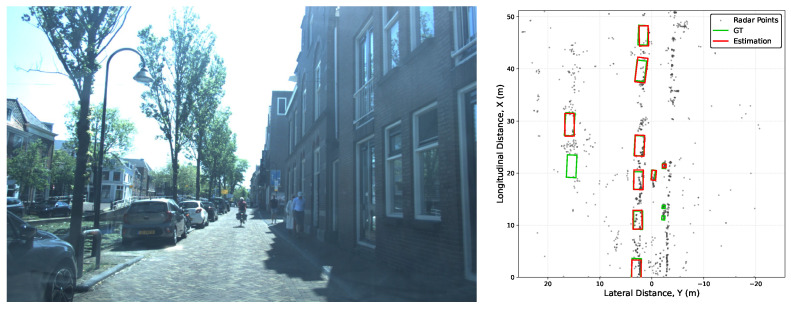

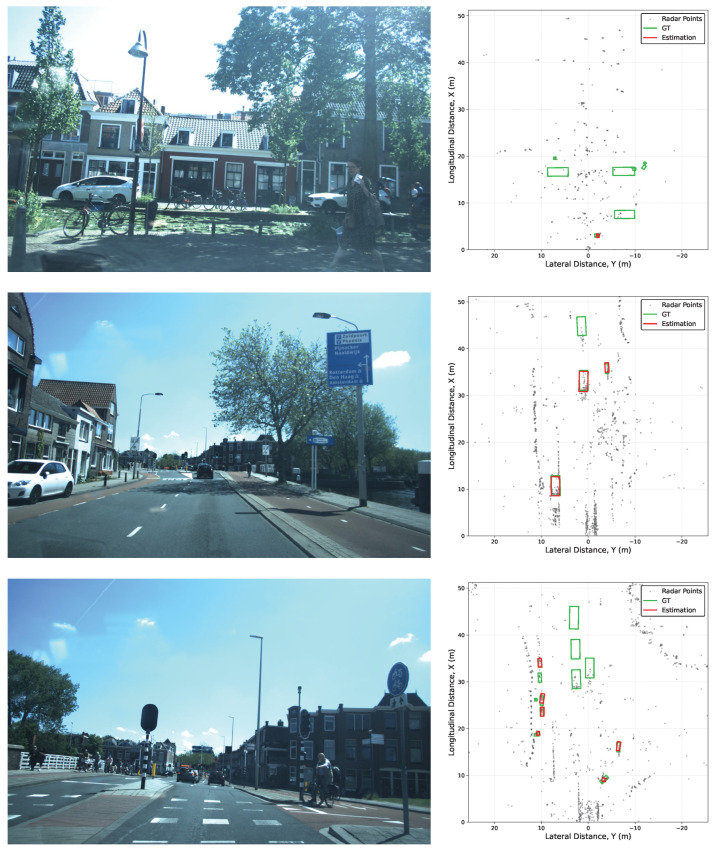
Qualitative detection results of SGE-Flow on the VoD dataset. Each row demonstrates the detection performance in a specific scenario, showing the camera scene (**left**) and the corresponding BEV radar point cloud (**right**). Red and green boxes represent the object Estimation and Ground Truth, respectively.

To evaluate the practical performance of the SGE-Flow framework, we present qualitative detection results across various urban scenarios in Figure 7. Each row in the figure displays a camera-view image (left) and the corresponding BEV radar point cloud (right). Our model successfully estimates the 3D bounding boxes of cars, pedestrians, and cyclists in complex and dynamic environments.

However, certain failure modes remain under specific conditions, primarily driven by the inherent sparsity of 4D radar point clouds. For instance, the second scenario exhibits noticeably lower point density compared to others, resulting in degraded estimation performance where only close-range moving pedestrians are successfully detected. Similarly, missed detections at longer ranges across various scenarios are largely attributable to this severe sparsity. Furthermore, the random distribution characteristic of mmWave radar points means that, unlike LiDAR, it struggles to capture the complete geometric contour of a target. Consequently, in complex environments where targets are in close proximity to buildings or other background objects, even short-range targets can be missed. A clear example of this is observed in the first scenario, where two pedestrians on the right side failed to be detected. These observations highlight the ongoing challenges in 4D radar perception and provide a clear direction for future research.

## 5. Discussion

The results indicate that the proposed modules contribute complementary benefits rather than redundant gains. VDC most strongly benefits pedestrians and cyclists, which is consistent with their higher motion variability and the sensitivity of Doppler features to displacement. DAD provides a more modest but stable improvement by alleviating vertical sparsity artifacts introduced by pillarization, while IFF delivers the largest overall gains by explicitly modeling temporal correlations that are otherwise lost in accumulated point clouds. Regarding random motion noise such as wind-shaken trees, since their movement across different pillars lacks target-level coherence and their Doppler signals typically manifest as local small-range fluctuations rather than continuous pillar-wise occupancy transitions, the Transformer encoder employed in this study can effectively distinguish structured target motion flows from random environmental fluctuations by capturing global context information. The ablation and augmentation studies also highlight an important practical constraint: radar velocity features are physically coupled to viewing geometry, so naive translations and rotations can degrade motion consistency even when they increase spatial diversity. This suggests that future work should prioritize physics-aware augmentation and domain-specific regularization. Finally, the mild performance drop observed with deeper IFF Transformer layer nums underscores a trade-off between model capacity and data sparsity, indicating that lightweight temporal modeling is preferable for 4D radar. These findings collectively support the use of compact, physically grounded feature engineering as a reliable path to real-time radar perception.

In addition, our current results are based on multi-frame point clouds, and ego-motion compensation has a substantial impact on performance; while the VoD dataset provides this step, extending the method to other millimeter-wave radar datasets may require extra effort to implement reliable compensation.

## 6. Conclusions

We have presented a streamlined feature extraction framework that redefines efficient 4D radar perception. We have demonstrated the potential of Doppler velocity and multi-frame temporal cues. This was achieved by directly addressing the intrinsic limitations of radar data: sparsity, the absence of tangential velocity, and motion-induced geometric distortion. Our experiments show that the Transformer-based Inter-frame Flow (IFF) module is key, significantly enhancing the representation of moving targets by capturing the temporal changes of pillar occupancy.

Architecturally, we have embedded these feature extraction modules seamlessly into the PointPillars voxelization process, avoiding complex, independent branches in favor of a streamlined design. This embedded strategy delivers a superior balance between precision and speed on the View-of-Delft (VoD) dataset: achieving a high 3D mAP of 53.23% while sustaining an inference speed of 72 FPS on an RTX 3090. Additionally, we integrate our IFF module into the MAFF-Net to achieve superior performance without significantly compromising the frame rate. This performance perfectly aligns with the stringent real-time demands of autonomous driving. Ultimately, our work serves as a proof of concept: 4D radar can indeed serve as a robust, primary sensor for autonomous perception without compromising real-time responsiveness.

## Figures and Tables

**Table 1:** Comparison of our SGE-Flow with state-of-the-art methods on the VoD validation set.

Method	Full Area AP				Driving Corridor/RoI AP				FPS (Hz)
	Car	Ped.	Cyc.	mAP (3D)	Car	Ped.	Cyc.	mAP (3D)	
PointPillars [4]	40.92	39.08	62.65	47.55	71.60	48.07	73.50	64.39	178 (3090)
SMURF [7]	42.31	39.09	71.50	50.97	71.74	50.54	86.87	69.72	30.3
RadarPillars [9]	41.10	38.60	72.60	50.70	71.10	52.30	87.90	70.50	179.1
MUFASA [31]	43.10	38.97	68.65	50.24	72.50	50.28	**88.51**	70.43	-
4DRadDet [32]	42.03	40.70	71.61	51.44	72.12	51.18	87.95	**74.15**	35.2 (4090)
SCKD [19]	41.89	43.51	70.83	52.08	77.54	51.06	86.89	71.80	39.3 (4090)
MAFF-Net [8]	42.33	46.75	74.72	54.59	72.28	**57.81**	87.40	72.50	28.7 (4090)
MAFF-Net [8] + Ours (IFF)	**43.72**	**47.13**	74.14	**55.00**	**79.98**	53.02	86.13	73.04	26.29 (3090)
Our SGE-Flow	41.23	43.46	**75.00**	53.23	71.81	53.66	87.10	70.86	72 (3090)

Note: “AP” stands for Average Precision, “mAP” stands for Mean Average Precision, “Ped.” stands for Pedestrian, and “Cyc.” stands for Cyclist. “MAFF-Net + Ours (IFF)” denotes the integration of our proposed Inter-frame Flow (IFF) module into the feature extractor of the MAFF-Net [8] to verify the modularity and generalization ability of the proposed component. Bold and underlined values represent the best and second-best performance, respectively.

**Table 2:** Ablation study of components on the VoD validation set.

					**Full Area AP**			
**Experiment**	**Baseline**	**VDC**	**DAD**	**IFF**	**Car**	**Ped.**	**Cyc.**	**mAP**
1	✓				41.17	36.11	66.28	47.85
2	✓	✓			**41.52**	40.53	70.48	50.84
3	✓	✓	✓		41.42	39.87	72.24	51.14
4	✓	✓	✓	✓	41.23	**43.46**	**75.00**	**53.23**
					**Driving Corridor/RoI AP**			
**Experiment**	**Baseline**	**VDC**	**DAD**	**IFF**	**Car**	**Ped.**	**Cyc.**	**mAP**
1	✓				**72.11**	41.93	86.83	66.96
2	✓	✓			71.80	50.31	84.70	68.94
3	✓	✓	✓		71.99	49.69	84.58	68.75
4	✓	✓	✓	✓	71.81	**53.66**	**87.10**	**70.86**

Note: “✓” indicates that the corresponding module or feature is enabled in the experiment. Bold values represent the best performance.

**Table 3:** Data augmentation methods and velocity selection.

$v_{r}$	$v_{r,\mathrm{comp}}$	Augment	Full Area AP				Driving Corridor/RoI AP			
			Car	Ped.	Cyc.	mAP	Car	Ped.	Cyc.	mAP
✓	✓	None	39.88	38.76	69.07	49.24	71.12	48.99	85.09	68.40
✓	✓	Obj. Trans.	41.12	38.92	68.39	49.48	71.70	49.27	83.06	68.01
✓	✓	Obj. Rot.	41.04	40.28	66.79	49.37	71.80	**53.96**	79.27	68.34
✓	✓	Glob. Rot.	40.81	40.13	72.19	51.04	71.27	48.90	**87.17**	69.11
✓		Glob. Rot.	40.19	41.94	71.34	51.16	70.67	52.45	85.07	69.40
	✓	Glob. Rot.	**41.23**	**43.46**	**75.00**	**53.23**	**71.81**	53.66	87.10	**70.86**

Note: “✓” indicates that the corresponding module or feature is enabled in the experiment. Bold values represent the best performance.

**Table 4:** Comparison of IFF and timestamp *t*.

Feature	*t*/IFF	Full Area AP				Driving Corridor/RoI AP				FPS
		Car	Ped.	Cyc.	mAP	Car	Ped.	Cyc.	mAP	
VDC	*t*	41.52	40.53	70.48	50.84	71.80	50.31	84.70	68.94	96.1
VDC	IFF	42.00	41.87	70.51	51.46	72.00	52.56	**85.42**	69.99	74.7
DAD	*t*	42.37	40.63	**71.76**	51.58	71.75	54.36	84.51	**70.21**	80
DAD	IFF	**42.40**	**43.79**	71.04	**52.41**	**72.04**	**54.80**	83.75	70.20	74.9

Note: Bold values represent the best performance.

**Table 5:** Transformer Layer Num in the IFF module.

Transformer Layer Num	Full Area AP				Driving Corridor/RoI AP			
	Car	Ped.	Cyc.	mAP	Car	Ped.	Cyc.	mAP
1	39.60	43.36	**75.19**	52.72	**71.95**	50.33	**87.33**	69.87
2	**41.23**	43.46	75.00	**53.23**	71.81	53.66	87.10	**70.86**
3	40.15	**43.47**	70.31	51.31	71.70	**54.09**	82.94	69.58

Note: Bold values represent the best performance.

## Data Availability

Publicly available datasets were analyzed in this study. The View-of-Delft (VoD) dataset is described in Ref. [11] and is available at: https://github.com/tudelft-iv/view-of-delft-dataset (accessed on 23 January 2026).

## Abbreviations

The following abbreviations are used in this manuscript:

4D	Four-dimensional
3D	Three-dimensional
mmWave	Millimeter-wave
LiDAR	Light Detection and Ranging
CNN	Convolutional Neural Network
BEV	Bird’s Eye View
VDC	Velocity Displacement Compensation
DAD	Distribution-Aware Density
IFF	Inter-frame Flow
KDE	Kernel Density Estimation
RCS	Radar Cross Section
mAP	Mean Average Precision
AP	Average Precision
FPS	Frames Per Second
Ped.	Pedestrian
Cyc.	Cyclist
MLP	Multi-Layer Perceptron
PE	Positional Encoding
FPN	Feature Pyramid Network
ROI	Region of Interest
GPU	Graphics Processing Unit
CUDA	Compute Unified Device Architecture

## References

[1] Zou Z., Chen K., Shi Z., Guo Y., Ye J. (2023). Object detection in 20 years: A survey. Proc. IEEE.

[2] Mao J., Shi S., Wang X., Li H. (2023). 3D object detection for autonomous driving: A comprehensive survey. Int. J. Comput. Vis..

[3] Zhou Y., Tuzel O. VoxelNet: End-to-end learning for point cloud based 3D object detection. Proceedings of the IEEE Conference on Computer Vision and Pattern Recognition (CVPR).

[4] Lang A.H., Vora S., Caesar H., Zhou L., Yang J., Beijbom O. PointPillars: Fast encoders for object detection from point clouds. Proceedings of the IEEE Conference on Computer Vision and Pattern Recognition (CVPR).

[5] Zang S., Ding M., Smith D., Tyler P., Rakotoarivelo T., Kaafar M.A. (2019). The impact of adverse weather conditions on autonomous vehicles: How rain, snow, fog, and hail affect the performance of a self-driving car. IEEE Veh. Technol. Mag..

[6] Bialer O., Jonas A., Jankirana M., Katz D., Moosazadeh S., Amram N. (2023). The rise of 4D imaging radar: A survey. IEEE Signal Process. Mag..

[7] Liu J., Zhao Q., Xiong W., Huang T., Han Q.-L., Zhu B. (2024). SMURF: Spatial multi-representation fusion for 3D object detection with 4D imaging radar. IEEE Trans. Intell. Veh..

[8] Bi X., Weng C., Tong P., Fan B., Eichberger A. (2025). MAFF-Net: Enhancing 3D Object Detection with 4D Radar via Multi-assist Feature Fusion. IEEE Robot. Autom. Lett..

[9] Roldan I., Pool E., Kooij J.F.P. RadarPillars: Efficient object detection from 4D radar point clouds. Proceedings of the IEEE International Intelligent Transportation Systems Conference (ITSC).

[10] Fan L., Wang J., Chang Y., Li Y., Wang Y., Cao D. (2024). 4D mmWave Radar for Autonomous Driving Perception: A Comprehensive Survey. IEEE Trans. Intell. Veh..

[11] Palffy A., Pool E., Baratam S., Kooij J.F.P., Gavrila D.M. (2022). Multi-class road user detection with 3+1D radar in the View-of-Delft dataset. IEEE Robot. Autom. Lett..

[12] Zheng L., Ma Z., Zhu X., Tan B., Li S., Long K., Sun W., Chen S., Zhang L., Wan M. TJ4DRadSet: A 4D radar dataset for autonomous driving. Proceedings of the IEEE International Intelligent Transportation Systems Conference (ITSC).

[13] Shi S., Guo C., Jiang L., Wang Z., Shi J., Wang X., Li H. PV-RCNN: Point-voxel feature set abstraction for 3D object detection. Proceedings of the IEEE/CVF Conference on Computer Vision and Pattern Recognition (CVPR).

[14] Yin T., Zhou X., Krahenbuhl P. Center-based 3D object detection and tracking. Proceedings of the IEEE/CVF Conference on Computer Vision and Pattern Recognition (CVPR).

[15] Xu B., Zhang X., Wang L., Hu X., Li Z., Pan S., Li J., Deng Y. RPFA-Net: A 4D RaDAR pillar feature attention network for 3D object detection. Proceedings of the IEEE International Intelligent Transportation Systems Conference (ITSC).

[16] Yan Q., Wang Y. (2023). MVFAN: Multi-view feature assisted network for 4D radar object detection. arXiv.

[17] Jia L., Zhang X., Zhao D., Zhang H., Wang Y., Liu Z. (2024). RadarNeXt: Real-time and reliable 3D object detector based on 4D mmWave imaging radar. arXiv.

[18] Lin Z., Liu Z., Xia Z., Wang X., Wang Y., Qi S., Dong Y., Dong N., Zhang L., Zhu C. RCBEVDet: Radar-camera fusion in bird’s eye view for 3D object detection. Proceedings of the IEEE/CVF Conference on Computer Vision and Pattern Recognition (CVPR).

[19] Xu R., Xiang Z., Zhang C., Zhong H., Zhao X., Dang R., Xu P., Pu T., Liu E. (2024). SCKD: Semi-supervised cross-modality knowledge distillation for 4D radar object detection. arXiv.

[20] Tan B., Ma Z., Zhu X., Li S., Zheng L., Chen S., Zhang L. (2023). 3D object detection for multi-frame 4D automotive millimeter-wave radar point cloud. IEEE Sens. J..

[21] Wang X., Li J., Wu J., Wu S., Li L. (2025). DADAN: Dynamic-Augmented and Density-Aware Network for Accurate 3-D Object Detection with 4-D Radar. IEEE Sens. J..

[22] Fu J., Liu J., Tian H., Li Y., Bao Y., Fang Z., Lu H. Dual Attention Network for Scene Segmentation. Proceedings of the IEEE/CVF Conference on Computer Vision and Pattern Recognition (CVPR).

[23] Li J., Yang L., Chen Y., Yang Y., Jin Y., Akiyama K. PillarDAN: Pillar-based Dual Attention Attention Network for 3D Object Detection with 4D RaDAR. Proceedings of the IEEE International Conference on Intelligent Transportation Systems (ITSC).

[24] Hu J.S.K., Kuai T., Waslander S.L. Point density-aware voxels for LiDAR 3D object detection. Proceedings of the IEEE/CVF Conference on Computer Vision and Pattern Recognition (CVPR).

[25] Li Z., Yao Y., Quan Z., Xie J., Yang W. (2022). Spatial information enhancement network for 3D object detection from point cloud. Pattern Recognit..

[26] Vaswani A., Shazeer N., Parmar N., Uszkoreit J., Jones L., Gomez A.N., Kaiser L., Polosukhin I. Attention is all you need. Proceedings of the Advances in Neural Information Processing Systems (NeurIPS).

[27] Lu F., Chen H., Wu J., Wang Y. (2026). Deep Sequential Learning for Pose Forecasting. IEEE Sens. Lett..

[28] Yan Y., Mao Y., Li B. (2018). SECOND: Sparsely embedded convolutional detection. Sensors.

[29] Qi C.R., Su H., Mo K., Guibas L.J. PointNet: Deep learning on point sets for 3D classification and segmentation. Proceedings of the IEEE Conference on Computer Vision and Pattern Recognition (CVPR).

[30] Lin T.-Y., Goyal P., Girshick R., He K., Dollar P. Focal loss for dense object detection. Proceedings of the IEEE International Conference on Computer Vision (ICCV).

[31] Wille L., Pangerl M., Keil T., Lopez D.S.G.P., Bierzynski K. MUFASA: Multi-view fusion and adaptation network with spatial awareness for radar object detection. Proceedings of the International Conference on Artificial Neural Networks (ICANN).

[32] Weng C., Bi X., Tong P., Eichberger A. 4DRadDet: Cluster-queried enhanced 3D object detection with 4D radar. Proceedings of the IEEE International Conference on Robotics and Automation (ICRA).

